# Prescriptions and proscriptions: moralising sleep medicines

**DOI:** 10.1111/1467-9566.12383

**Published:** 2015-11-20

**Authors:** Jonathan Gabe, Catherine M. Coveney, Simon J. Williams

**Affiliations:** ^1^ Centre for Criminology & SociologyRoyal HollowayUniversity of London; ^2^ Centre for Global Health PolicyUniversity of Sussex; ^3^ Department of SociologyUniversity of Warwick

**Keywords:** sleep, pharmaceuticals/pharmaceutical companies, drugs/medication, UK

## Abstract

The pharmaceuticalisation of sleep is a contentious issue. Sleep medicines get a ‘bad press’ due to their potential for dependence and other side effects, including studies reporting increased mortality risks for long‐term users. Yet relatively little qualitative social science research has been conducted into how people understand and negotiate their use/non‐use of sleep medicines in the context of their everyday lives. This paper draws on focus group data collected in the UK to elicit collective views on and experiences of prescription hypnotics across different social contexts. Respondents, we show, drew on a range of moral repertoires which allowed them to present themselves and their relationships with hypnotics in different ways. Six distinct repertoires about hypnotic use are identified in this regard: the ‘deserving’ patient, the ‘responsible’ user, the ‘compliant’ patient, the ‘addict’, the ‘sinful’ user and the ‘noble’ non user. These users and non‐users are constructed drawing on cross‐cutting themes of addiction and control, ambivalence and reflexivity. Such issues are in turn discussed in relation to recent sociological debates on the pharmaceuticalisation/de‐pharmaceuticalisation of everyday life and the consumption of medicines in the UK today.

## Introduction

Sleep is not just a personal or political matter but a moral one, including the very meanings we accord sleep and the ways we manage it in our everyday/night lives. In this paper we take a closer look at these issues through the multiple meanings and moral dimensions of sleep medications in everyday/night life. Our paper, in this regard, is located at the intersection of newly emerging social scientific scholarship on sleep matters (Henry *et al*. 2013, Williams 2005, 2011, Williams *et al*. 2013, Williams and Wolf‐Meyer 2013), wider sociological debates on the medicalisation and pharmaceuticalisation of life (Abraham 2010, Bell and Figert 2012, Gabe *et al*. 2015, Williams *et al*. 2011), and other work in medical sociology (Lumme‐Sandt and Virtanen 2002, Pound *et al*. 2005) and cognate fields such as science and technology studies (STS) (Coveney 2011, Oudshoorn 2008, Webster *et al*. 2009) on the meanings, cultural scripts and uses of pharmaceuticals and other socio‐technological objects.

Redefining sleep as a medical problem requiring a pharmaceutical intervention is a contentious issue (cf. Anthierens *et al*. 2007, Kripke 2006, PCT 2011, Rogers *et al*. 2007). Sleep medications get a ‘bad press’ due to their potential for dependence and other side effects, including studies reporting increased mortality risks for long‐term users (Kripke *et al*. 2012). Medically, the long term use of hypnotics is not advised due to adverse side effects, including drowsiness, confusion and cognitive and psycho‐motor impairment, effects which have been associated with fractures, falls and road‐traffic accidents (Buysse 2013, Siriwardena *et al*. 2008,), in addition to problems of tolerance and dependence. In 2004, NICE (National Institute for Health and Clinical Excellence) in the UK issued guidance to physicians about these risks and advised against use for over 4 weeks, and only for severe insomnia. Previously similar advice had been issued by the Department of Health and Social Security in the 1980s about the dependence potential of benzodiazepines, including those marketed as sleeping tablets (Gabe and Bury 1988), yet in 2013, over 9.7 million hypnotics were still dispensed in the UK including 6.3 million ‘z drugs’, primarily zopiclone and 2.9 million benzodiazepines (HSCIC 2014). Approximately a quarter of these prescriptions were for four weeks or more, contrary to guidance from NICE (2004).

A small number of sociological studies have looked at patient experiences of taking sleeping pills. These studies found that both the prescription of benzodiazepine hypnotics in primary care and consumption of these medicines in daily life are highly moralised issues (Gabe and Lipshitz‐Phillips 1982, 1984, Gabe and Thorogood 1986, North *et al*. 1995, Venn and Arber 2012). Ambivalence to using these medicines emerges as a common theme across different patient groups where the benefits or need to medicate sleep is juxtaposed with more negative feelings towards hypnotic medication (Gabe and Lipshitz‐Phillips 1982, 1984, Gabe and Thorogood 1986). For instance a recent study looking at older people's experience of taking sleep medications found that hypnotic use was often viewed as being morally inappropriate – ‘an unnatural interference into a natural state’ – and associated with loss of control and addiction (Venn and Arber 2012). Similarly, the women in earlier studies (Gabe and Lipshitz‐Phillips 1982, 1984, Gabe and Thorogood 1986) expressed a strong dislike of taking drugs of any kind, and while accepting that the drug might do them some good also expressed fears of addiction and dependency.

Social scientific research on pharmaceuticals more generally has, amongst other things, drawn attention to how pharmaceuticals are understood and used in everyday life. Pharmaceutical technologies are socially embedded (Cohen *et al*. 2001) and their use shaped by cultural repertoires, social relationships, the medical condition being experienced and the identities of their consumers (Dew *et al*. 2015, Lumme‐Sandt *et al*. 2000, Rose, 2007, Webster *et al*. 2009). They are thus imbued with not only technical meanings relating to their biomedical functions but also with strong social and cultural scripts for how they should be used (Hodgetts *et al*. 2011). Users are increasingly recognised as being knowledgeable and reflexive actors, assessing the risks and benefits and making informed choices about medicine use drawing on what Webster *et al*. (2009) call a ‘lay pharmacology’ about safety, efficacy and side effects. Such choices are sometimes made in consultation with professionals and sometimes not (Will and Weiner 2015).

Modifying medicine treatment regimens without prior discussions with medical professionals is common place. For example, in a review of qualitative studies Pound *et al*. (2005) reported that dosages were generally decreased by patients in their attempts to maintain control of medications and medicines were often supplemented or replaced with non‐pharmacological treatments. Pharmaceutical drugs are thus but one part of a larger assortment of medical and non‐medical technologies, devices, discourses and talking therapies aimed at modulating physical, behavioural, psychological and emotional states. Dew *et al*. (2013) argue that people ‘hybridise these wellness practices’, assimilating different forms of knowledge and expertise and recombining them in relation to their own understandings in the enactment of their daily routines and relationships. The availability of such non‐medical technologies thus provides opportunities for de‐pharmaceuticalisation at the lay or life world level.

In this way, we can think about hypnotic medicines as consumer goods that are personalised and reconfigured in the home. They are consumed within socio‐technical networks that give meaning to their use and non‐use. Through our interactions with medicines in daily life these biomedical objects are translated into social objects that carry biographies, personal and shared meanings, thus becoming ‘socio‐pharmacological objects’ (Hodgetts *et al*. 2011). Different meanings are given to medications in daily life and these meanings are important to understand the variations in how medicines are used. Hence, not only the practicality of taking ones medication, but the morality of medicine use, and associated ideas about ‘good’ and ‘bad’ behaviour, is an important consideration.

Lumme‐Sandt *et al*. (2000), in their study of the oldest old (aged 90 or over) in Finland found that their respondents called primarily on a moral repertoire when talking about medications (including sleeping pills). They presented themselves as moral and responsible drug users by explaining the ‘objective reasons’ for their use, playing down the extent of such use and comparing it favourably with assumed level of use by others. In a similar vein Dew *et al*. (2015), in their study of households in New Zealand, reported that respondents developed and articulated four distinct repertoires about the moral meaning of medications: a disordering society repertoire where pharmaceuticals evoked a society in an unnatural state which required active resistance; a disordering self repertoire where drug use indicated a moral failing of the individual and a lack of control; a disordering substances repertoire involving a threat to a person's physical or mental state but where medicine use could be justified on the basis of a cost benefit assessment and the importance of acting responsibly; and finally a re‐ordering substances repertoire where drug use was associated with a restoration of function in line with the advice of professionals.

This focus on moral judgements can be located in wider concerns to understand health and illness in terms of the accounts people give, the vocabularies they draw on and the contexts in which these accounts are constructed (Backett 1992, Radley and Billig 1996). This means that morality is not viewed as located in people's heads but instead is an aspect of the embodied interactional practices that people engage in as members of society (Sayer 2011, Turowetz and Maynard 2010). Such practices can involve expressing approval or disapproval of others or presenting oneself as a moral being (Sayer 2011).

In this paper, as a further contribution to these moral matters concerning medicines in general and sleep medicines in particular, we aim to explore the ways in which the use/non‐use of prescription hypnotics is understood and negotiated in daily life and how this is implicated in moral discourses about medicines. Drawing on focus group data we analyse collective views and experiences of hypnotic use across a range of social contexts, paying attention to the moral dimensions of respondents’ talk and the repertoires they draw on to justify and legitimate hypnotic use/ non‐use in the management of sleep problems. By ‘repertoire’ we mean a relatively coherent system of meanings for ‘characterizing and evaluating actions, events and other phenomena’ (Potter and Wetherell 1987: 149). It is important to recognise that ‘the same person may use different repertoires in different contexts and for different functions’ (Lumme‐Sandt *et al*. 2000: 1845). How users engage with hypnotics in their daily lives moreover, we argue, is an important yet under‐researched dimension of understanding the dynamics of pharmaceuticalisation in an era where users of medicines, sleep related or otherwise, are increasingly active in the management of their own health and illness.

## Methods

Data were collected as part of a wider study looking at Medicated Sleep and Wakefulness in the UK since 2000, funded by the UK Economic and Social Research Council. Following ethics approval from the National Health Service in England we held 23 focus groups (99 participants), between 2012 and 2014, with people who might be expected to have particular views and experiences of sleep management. This included those currently taking hypnotics prescribed in a primary care setting, people who have been diagnosed with a sleep disorder (narcolepsy, sleep apnoea, insomnia), and general population groups including students, parents of young children, ambulance service staff (including technicians and paramedics), academics, lawyers and retired people living in sheltered housing. We purposively selected these groups in order to explore diversity in experiences of and attitudes towards sleeping pills rather than for representativeness.

Research participants were recruited in a number of ways. General practice patients were invited to participate by their GP, on the basis of having received a prescription for sleeping tablets. Those attending sleep apnoea clinics and narcolepsy clinics were invited to contact the researchers if they were interested in being part of the research by the clinician in charge of the clinic. Paramedics were recruited through advertisements in the local Trust newsletter and posters sent to ambulance stations, while students were invited through the local university student union, departmental student lists and personal contacts. Parents of young children were recruited through local parent and toddler groups. The focus groups made up of academics and lawyers were formed through personal contacts. Those who lived in a retirement complex were invited to participate via a gatekeeper who lived on site, following agreement from the manager and the residents’ committee. To our knowledge none of those who accepted our offer to take part had been diagnosed as suffering from dementia, which would have had a significant impact on their sleep and their understanding of it.

The resulting 23 focus groups were quite diverse in terms of age, with approximately half the sample being 45 years of age or over. Around 60 per cent of participants were female and around 90 per cent identified as of white British or Irish ethnicity. Just over half the sample had, or had previously had before retirement, a higher managerial or professional job (See Table 1).

**Table 1:** Medicated sleep: participant demographics

Focus groups	Number of participants	Gender (M/F)	Age range	Ethnicity	SES	Number taken hypnotics
Academics 2 groups (AFG1–2)	8	5M 3F	25–54	7 WB/Irish 1 mixed (White & Asian)	Higher managerial & professionals	2
Ambulance service staff 3 groups (ASFG1–3)	9	5M 4F	25–54	9 WB/Irish	Higher managerial & professionals	3
Lawyers 1 group (LFG1)	3	2F 1M	35–44	2 WB/Irish 1 White (other)	Higher managerial & professionals	2
Narcolepsy patients 2 groups (NFG1–2)	13	7F 6M	18–74	12 WB/Irish 1 BB (African)	5 Intermediate 4 Technical & craft 1 Not disclosed 1 Unemployed 1 Student 1 Higher managerial & professionals	3
Parents of young children 2 groups (PFG1–2)	10	6F 4M	25–44	8 WB/Irish 1 Asian or AB (Pakistani) 1 White (other)	6 Higher managerial & professionals 3 Intermediate 1 Technical & craft	1
Primary care patients 3 groups (PCFG1–3)	12	6F 6M	45–85+	12 WB/Irish	6 Higher managerial & professionals 1 Intermediate 3 Technical & craft 2 Not disclosed	12
Retirement complex 3 groups (RFG1–3)	15	14F 1M	65–85+	15 WB/Irish	9 Higher managerial & professionals 3 Intermediate 3 Technical & craft	10
Sleep apnoea patients 3 groups (SAFG1–3)	13	8M 5F	45–74	13 WB/Irish	10 Higher managerial & professionals 1 Intermediate 2 Technical & craft	4
Students 4 groups (SFG1–4)	16	11F 5M	18–44	10 WB/Irish 2 White (other) 1 Mixed (other) 1 Asian 2 Chinese	Students	4
Totals	99	41M 58F	18–24 (15) 25–34 (18) 35–44 (15) 45–54 (14) 55–64 (10) 65–74 (11) 75–84 (12) 85+ (3) Not disclosed (1)	88 WB/Irish 4 White (Other) 2 Chinese 1 Black British 2 Asian/ Asian British 2 Mixed	52 Higher managerial & professionals 14 Intermediate occupations 12 Technical & craft 17 Students 1 Unemployed 3 Not disclosed	41

Focus groups were moderated by two of the research team, with one leading and the other taking notes. In each focus group we asked participants to discuss how they managed sleep problems in their daily lives and what they thought the appropriate role of pharmaceuticals was in their management practices. Forty one participants, across 21 of the focus groups, disclosed current or previous use of prescription hypnotic medications (see Table 1). Of these just over half were members of the primary care or sheltered housing focus groups. Experience of use ranged from one short course of hypnotics to several years or in some cases, decades of use (up to 40 years). The medications used included benzodiazepines, Z drugs (e.g. Zopiclone) and other forms of sedative such as Melatonin, Sodium Oxybate (Xyrem) and sedative antidepressants.^1^, ^2^ The paper focuses particularly on those who said they were current or previous users of prescription sleep medicines and how they interacted with those (n = 58) who said they had never used these medicines.

Focus groups were used as the means of data collection in order to explore people's views about, and experiences of using hypnotics and issues around the use of sleeping pills in daily life. Focus groups were audio recorded and transcribed. Analysis of the transcripts was facilitated using the qualitative data analysis software package NVivo 10 (QSR International, Brisbane). We took an inductive approach to data analysis which involved reading and re‐reading the transcripts, grouping data extracts together based on their main themes and developing a coding frame based on these emergent themes to identify major topics and issues. Codes and themes relating to major issues were discussed between the authors for purposes of reliability and validity. These were used to develop an interpretative analysis of the meaning of hypnotic use and draw out the implications for the research questions outlined above.

Subsequently each focus group member was given an identifying code, indicating the type of focus group they had participated in, the number of the group and the gender of the participant, and the sequence in which they first spoke in the focus group (for example PCFG1F1 indicates primary care focus group 1, Female 1). Focus groups can be distinguished from one‐to‐one interviews as during a focus group participants are encouraged to engage with one another and this interaction between participants is included as research data (Kitzinger 1994). This methodology generates data not only about what people think about a certain issue, but also draws out the moral dimensions of how they think about it and why they think as they do. Using focus groups as a research tool, therefore, enabled us to: explore how people talk about sleep and the management of their sleep problems together; assess how ideas are formed and decisions are made regarding the ‘appropriate’ role of sleep medications in such management strategies and how they evaluate their own relationships with sleeping pills.

Below we consider six distinct repertoires about hypnotic use. These are constructed drawing on the cross‐cutting themes of addiction and control, ambivalence and reflexivity. These crosscutting themes are considered in the discussion.

## Moral repertoires: themes and tensions

In focus group discussions participants drew on a range of moral repertoires which allowed them to present themselves and their relationships with hypnotics in different ways. We can identify six repertoires about hypnotic use in this regard – the ‘deserving’ patient, the ‘responsible’ user, the ‘compliant’ patient, the ‘addict’, the ‘sinful’ user and the ‘noble’ non‐user. These are discussed in turn below.

### The ‘deserving’ patient

Participants were asked if they had ever been to see a doctor about their sleep problems or if they had ever taken any prescribed medication for sleep. The answers to these questions tended to be given in the form of a series of short monologues, where each member of the group took turns to share their story, allowing each of them to explain and justify why they were taking sleeping pills or had taken this type of medication in the past. Typically, the other members of the group would listen to each story and wait for their turn, occasionally expressing their empathy by offering reassuring statements, agreeing with and backing up the experiences described by others. These accounts were very rarely questioned or challenged.

For example, the following brief accounts were provided in turn by four members of a primary care focus group, (two male and two female, aged 55–85+):

PCFG1M2 The reason I had [sleeping pills] was because I had a bad accident, several years ago and the particular hospital I was in said ‘something to help you sleep and when you come out just go along and see your doctor for a prescription and continue’ […] Simple as that.

PCFG1M1 Well, when I approached my doctor about depression, brought on with my wife going to hospital for a major operation, as far as I was concerned, lack of sleep was driving me into depression. Of course I was worried about my wife, extremely, but if there was one factor that could help me through it all was the sleep.

PCFG1F2 And the lack of sleep means you can't actually cope, doesn't it, with what you need to cope with, I think.

PCFG1M1 That's right. […].

PCFG1F1 With me it was I didn't sleep at the time my husband died. He died suddenly and I just struggled along for a number of years but various problems that I had, not really coping all that well at the time. Doctor gave me […] a prescription for three. He said ‘take one of these every second day and see how you go on’. Well, it was lovely, absolutely lovely but I was back within three days ‐ ‘can I have some more?’ So, it's gone on from then, really.

Every one of the participants who had taken prescription medication to help them sleep acted to present themselves as deserving of a pharmaceutical solution for their sleep problem. Therefore, the deserving patient repertoire was found across all of our focus groups. A typical way of doing this was to present themselves as in need of sleeping pills, using this as moral justification for their medication use.

As shown in the extract below, many of the respondents described complex, enduring and severe health and social problems that contributed towards poor sleep. These ranged from cancer, pain, anxiety, depression and stress to social and relational problems such as bereavement, social isolation, redundancy, financial problems, relationship breakdowns, and caring for ill family members. Some clearly attributed these as causal factors in the development of their sleep problems. Others described their problems with sleep as existing alongside and often exacerbating or being exacerbated by other health conditions and social issues they had faced in their lives. Through discussion of their health, life circumstances and interactions with medical professionals, the participants depicted themselves as deserving patients who had had their sleep problems medically recognised and pharmaceuticals prescribed as a necessary medical treatment, which in turn acted to validate them as deserving patients who were justified in taking sleeping pills:

SAFG2F1 Not sleeping? Well, all the doctors said it was because of the stressful life I had, and I had as I say a very sick child and my husband was away a lot, and a very, very stressful job, and so I went to a doctor and I just said, you know ‘*I need something* [emphasis added] to get me to sleep’ so they gave me some tablets. (Sleep Apnoea Focus Group Two, female, aged 55– 64)

It was common for participants to talk about other medications that they took alongside (or instead of) sleeping pills. These included antidepressants and pain killers that have a sedative effect and non‐sedative medications they took for a range of other conditions such as hypertension and diabetes. Thus they presented themselves as in need of a range of medical treatments and their use of sleeping pills as just one part of their medicated self.

These illness narratives allowed participants to present themselves as in distress and in need of help, to justify why they had sought medical advice and legitimate why they were taking, or had taken medications for sleep.

### The ‘responsible’ user

In addition to presenting themselves as deserving of and in need of medication, the respondents typically presented themselves as vigilant in self‐monitoring their use of sleeping pills. It was common for participants to construct an image of themselves as being responsible users who used their medication appropriately. The ‘responsible user’ repertoire was drawn on by academics, ambulance service staff, primary care patients, retired persons, sleep apnoea patients and students. The responsible user is one who is knowledgeable about their medication and its effects and is reflexive about their use. They are actively concerned about becoming dependent on or addicted to sleeping pills and describe taking steps to minimise this. They were ambivalent about their use of hypnotics, indicated they knew about the medication they took and its effects on them, questioned medical expertise and advice and made choices which they felt were in their best interest, drawing on their own ideas about appropriate uses of medication in daily life. For instance, these participants told stories about asking their doctor for their dosage to be reduced if they felt they were becoming reliant on the medication and altering their pharmaceutical regimens outside of medical authority to safeguard against dependency or addiction. They did this in various ways, for example, by using medication intermittently or as a last resort when they were extremely fatigued and ‘could not go on’ without sleeping, reducing the dose by cutting pills in half or into quarters, and substituting their medicine for a herbal remedy. Typically, medicated sleep was classed as inferior to ‘natural sleep’ – or sleep without medication, with the latter idealised.

In the exchange below we see an example of such self‐surveillance and self‐governance. The participants present themselves as being responsible for their own drug use, carefully monitoring their levels of consumption and acting to minimise their use of the sleeping pills, of their own volition:

RFG3F3 What frightens me, I had a hip operation and I have to take some medication to get me to sleep at night because of the pain. The trouble is, after a few nights, you're beginning to rely on it. And that's frightening. And so you have to be, as [RFG3M1] said, self‐controlling, and try to control the drug, and gradually lessen it.

RFG3F2 That's why you cut it down if you can.

RFG3F3 If you can. (Residential Focus Group, two female participants, aged 75–84)

In cases where there was only one person in the focus group who had taken sleeping pills in the past, the participant could find their need to use sleep medication being challenged by other members of the group, pushing them to justify and explain further why they had turned to sleeping pills, to demonstrate that they were not only deserving of sleeping pills but also responsible in their use of them.

### The ‘compliant’ patient

When asked further questions about how they took their medication a small number of participants went on to present themselves as not only a deserving patient, but also a compliant one – someone who deferred to medical expertise, followed medical advice to the letter and respected medical authority. Talk of this kind was found across the three primary care patient groups and also in the focus groups held with retired persons and ambulance service staff. These participants described taking their medication in line with the dose and frequency recommended by their doctor. Presenting an image of being a compliant patient meant that in some cases they accepted that their use of this medication was ‘for life’; in other cases, they explained how they were trying to cut down or stop using hypnotic medication because their doctor had told them to do so:

PCFG1M1 I was with Dr [name] at the time and she told me when she prescribed them ‘you realise that you are on these for life?’ I said ‘yes, if they do what they say on the packet’ and it does. I just take the recommended dose and that's it. (Primary care focus group 1, Male participant, aged 55–64)

Compliance with medical advice was also a frequent theme in focus group discussions amongst those who had never taken sleeping pills or seen a doctor about sleep problems. In such cases, it was common for participants to assert that they would take this type of medication, if it was prescribed to them and its use advised by their doctor.

Although presenting their use of sleeping pills as complying with medical advice, participants were also at times somewhat critical of this and expressed a high level of ambivalence about their use of these medicines. They acknowledged that sleeping pills helped them to get some sleep but at the same time talked about their unpleasant side effects, occasions when they did not work, and in some cases, feelings of guilt and embarrassment for their continued use. Despite presenting themselves as compliant patients, when asked directly about altering treatment regimens it was common for these patients to admit that they had increased the dosage of their medication at some point, but they stressed that it was a one‐off, it was not worth it, or that it did not do anything for them:

Moderator You take them every day?

PCFG2M2 I take them every night, yeah, about nine o'clock at night, 8.30 at night. I see them as placebo.

Moderator Why do you still take them, then?

PCFG2M2 It's like I'm clutching at straws, I suppose, yeah.

PCFG2M1 Safety blanket.

PCFG2M2 Yeah, I suppose it's like, it serves its need.

Moderator So, do you set them up in advance and take them as part of your routine?

PCFG2M2 I do, yeah […] they go out before … antidepressant for the morning time. So, I went from being I thought I was physically fit, all of a sudden I went up to four prescriptions, four tablets a day, medication, I don't know what happened to myself.

Moderator Do you actually vary the number you take?

PCFG2M2 No, it's only one. I have tried more than one just to see if it helps but no […] I've done it on a few occasions. I limit myself when I go to sleep, I take two tablets but it's not much effect.

Moderator Doesn't make that much difference?

PCFG2M2 No. They only give you a month's supply now so they do. They know, they keep track on how many you use, so they know, so you couldn't take them willy nilly. (Primary care focus group 2, two male participants, aged 45–64)

The stigma surrounding use of sleeping pills was apparent throughout the data. We asked participants if they had ever taken any medication for sleep, both on a short demographic questionnaire and during the focus group discussion. On occasion, when participants had said they had not taken sleeping pills on the questionnaire, it came out later in the discussion that in fact they had previously taken this type of medication.

In these cases, adopting the sick role and the identity of a compliant patient functioned to allow them to legitimate their previous use of sleeping pills as following doctor's orders. They had a medical need which was addressed by a medically prescribed treatment, which they took as advised by their doctor.

### The ‘addict’

Typically, participants did not orientate themselves towards being an addict and were critical of being mis‐represented as one. The addict repertoire was drawn upon by participants across all focus groups with the exception of narcolepsy patients, who are on medication for life, regarding this in terms of medical necessity rather than through an addiction frame, and parents of young children, who were more concerned with short term effects of medication on their daily functioning rather than possible effects of long term use. Addiction was associated with escalating use and a loss of control over ones use of medication and oneself. Long term users of hypnotics in particular strove to distance themselves from being seen as an addict, by depicting themselves as deserving, responsible or compliant, as discussed above. There was a general reluctance to disclose any information that might suggest they were addicted to sleeping pills. However, acknowledging long term use of these pills and/or a need to take them in order to sleep could lead to feelings of ambivalence about being seen as an addict and, as seen in the extract below, to participants questioning whether they could be classified as an addict or not:

PCFG3M1 I remember vaguely I think talking about sort of getting addicted, and I think he [GP] said you know, you want to make sure you're not taking them too often. But I'm almost of the philosophy that if he doesn't say anything, then it's okay. And I find it very comforting that I've got them there for when I need them. But I do very much try and watch it myself.

Moderator Is addiction an issue in your mind?

PCFG3M1 No, I mean, it's an issue in the fact that I don't want to become addicted, but I honestly don't think I am addicted. There's the occasion I've forgotten about them and absolutely got worried, but it hasn't been a disaster. If I was addicted to them, it really would cause problems.

PCFG3F1 I don't perceive myself as having an addictive personality. I think I could have been addicted in situations that have been offered to me in the past, I could have got myself addicted […] So I don't perceive myself as being an addict. However, I don't sleep without them, so does that make me an addict? (Primary care focus group 3, one male and one female participant, aged 55–64)

The occasions on which individuals acknowledged their vulnerability towards addiction, dependency and tolerance tended to be when talking about previous rather than current medication use. In these cases, as discussed in the above sections, participants told stories about how they recognised that their use was getting out of control and how this acted as a trigger for them to attempt to reduce their reliance on medication or stop taking the medication altogether.

In some cases, the participant's doctor had spoken to them about reducing or withdrawing the medication, or told them outright that the medication they were prescribing should not be used long term, citing risks of the addictive potential of the drugs. Doctors telling their patients that they should not be taking these tablets long term whilst at the same time continuing to issue them a prescription can appear conflicting. On the one hand, it conveys the moral impression to patients that their prolonged use of these substances is ‘wrong’ and the people who use them long term could become ‘addicts’ rather than legitimate patients. On the other hand, they are still having their problem medically validated and pharmaceuticalised by being issued a prescription for sleeping pills. This can result in divided emotions amongst patients, where feelings of guilt and embarrassment about their continued use of this medication coexist with their belief that they still have a legitimate need for it:

PCFG1F2 I was told only the other day, you become addicted to this, you must try and stop taking them. I said, I've been trying to stop taking them for a very long time, but I don't sleep if I don't take them.

PCFG1M1 Do you feel guilty about taking the sleeping tablets?

PCFG1F3 It isn't guilt, really, it's just that I suppose I was told at the beginning ‘you shouldn't take them for long, it will help you over this spell’ and then ‘it will help you over this’ and it's because I'm told they are addictive and, yes, I feel I shouldn't be taking pills. Other people manage to sleep every night and get through a night without pills.

PCFG1F2 But they're not ill are they?

PCFG1M2 I think the word addictive is one of these words that you don't really want in your …You are eating the forbidden fruit.

PCFG1F3 But I don't particularly want to be taking pills.

PCFG1M3 I feel guilty to ask for them.(Primary care focus group 1, 3 male participants and 2 female participants, aged 55–85+)

In such cases, it was all the more evident how respondents strove to portray themselves as deserving patients and their use of sleeping pills as appropriate. They attempted to distance themselves from the idea that they might be an addict by emphasising how they did not want to take pills but their continued use was necessary for them to get some sleep, which they needed in order to function in daily life. As discussed previously, it was typical for participants to describe the strategies of self‐surveillance and self‐governance they adopted precisely for this very reason: to protect themselves from becoming an addict.

### The ‘sinful’ user

When discussing modifications they made to their treatment regimens participants also discussed a variety of medically unsanctioned practices that they had been ‘guilty of’. These included increasing their dose of medication, stockpiling medicines, using medications prescribed for other reasons or to other people to help them sleep, and circumventing medical authority in order to obtain sleeping pills that they could not get on prescription, either because their doctor refused to prescribe or because the medication (such as melatonin supplements) was not readily available in the UK. Talk of this kind was found in the focus groups held with primary care patients, sleep apnoea patients, retired persons, students and lawyers. These activities were referred to by some of the participants as ‘wrong’, ‘being naughty’ or ‘sinning’. In ‘confessing’ these practices participants acknowledged that they were going against medical advice, but were able to ‘forgive’ themselves due to being in additional need or finding themselves in exceptional circumstances, such as when experiencing ‘a bad night’ or finding themselves in unfamiliar surroundings. At the same time they were still careful to present themselves as deserving and responsible, reflexive users who remained in control of their use of sleeping pills. Consider for example, the following exchange between three members of a primary care focus group:

PCFG1M3 I have sinned occasionally. And done that when there has been a big day, a long travelling day or doing something and I haven't felt any side effects. I've been quite happy.

PCFG1M2 I must admit, the most I've ever had is two of these things in a night, but I haven't had any side effects, perhaps that's unusual…

PCFG1M1 Some nights it's a real bad night […] and I know I should only have one tablet but ‐ it might happen about once every two or three months […] I might end up having 1.5 or two tablets, almost, but I've thought ‘well, I've got to get some sleep some time’.

PCFG1F1 I think we all know the dangers of being over‐sedated, and the dangers that there are of falling over the cat or something and breaking your leg or something. There is that danger, of course there is that danger, but occasionally if you have an extra one, if you've got something coming up you've just got to forgive yourself and get on with your life. (Primary care focus group 1, 2 male participants and one female, aged 55‐64)

In the next extract, participants from a residential focus group discuss how they shared tablets when not able to get them from the doctor:

Moderator Because it's so difficult to get sleeping tablets, have you ever shared them with each other?

RFG1F4 When we run out.

RFG1F2 Be careful what you say [RFG1F4].

Moderator Don't worry!

RFG1F2 She is inclined to slide me a few Temazepam.

RFG1F4 If she was running out, I'd say ‘have some of mine’.

RFG1F7 See you shouldn't do that.

RFG1F4 But you were running out of your prescription.

Moderator Anyone else share tablets?

RFG1F2 We don't share any tablets now; it was just if we were running out.

RFG1F4 Only because we couldn't get them.

RFG1F2 Because we couldn't get them. (Residential focus group 1, 3 female participants, aged 75–84).

It is clear that they were usually secretive about this and knew that it was not considered an appropriate way to obtain medication.

### The ‘noble’ (or ‘virtuous’) non‐user

A prevalent theme in our data, found predominately in discussions of medicating sleep amongst non‐users and previous users of hypnotics (ambulance service staff, academics, lawyers, parents of young children, retired persons, sleep apnoea patients, students), was the rejection of pharmaceuticalisation of sleep. This was tied in with the belief that it was a sign of moral strength not to rely on artificial props – a form of pharmaceutical Calvinism (Klerman 1972). The data we draw on here comes from participants who disclosed sleep problems or difficulties but were opposed to a pharmaceutical solution for them. Different reasons were given for the rejection or resistance to such pharmaceuticalisation. Some saw their sleep problems as not severe enough or not deserving of a medical solution. Difficulties sleeping were positioned as something people *should* be able to deal with themselves. In such discussions, respondents typically took an anti‐medication stance and saw the use of hypnotics as ‘giving in’ to strong medication, ‘taking the easy route’ or a ‘lazy’ way to deal with ones problems, with a view of medicated sleep being ‘unnatural’.

Others did not want to take sleeping pills, even in times of legitimate medical need (e.g. they had been prescribed sleeping pills but decided not to take them). In some cases this was due to past experiences of taking sleeping pills and experiencing negative side effects from the drugs. In others, it was because they worried about the effects the drug might have on them, including addiction, loss of control over their situation or impairing their ability to function.

For example, in the extract below from a Residential focus group, a male participant (RFG3M1) expresses his resistance to pharmaceuticalisation of sleep. The discussion in this particular group was moralistic and anti‐medication, although several of participants had taken sleeping pills in the past. Although initially presenting himself as a non‐user, later in the focus group this participant disclosed previous use of sleeping pills over a short period of time. He presented himself as a deserving patient at the time, but did not like the effect the medicine had had on him and rejected it subsequently, defying medical advice. Others in the group echoed his concerns about loss of control, and the side effects of medication and mentioned their own attempts to reduce the amount they took or their success in stopping taking the medication altogether.

RFG3M1 50 years ago I had a serious illness, which lasted about 3 years. And I was admitted to hospital initially. And one of the things, I was in a lot of pain and I wasn't sleeping. So I was prescribed sleeping pills, which I took for two nights and woke up with a hangover the following morning. I was asked by the consultant why I wasn't taking my medication, because I thought I'm going to refuse to take it. He agreed because I was adamant that I didn't want the hangover in the morning. And I've never ever had any aid to sleeping in the form of a sleeping tablet since that one occasion […] Unnatural sleep is hypnotic sleep. I think that induces adverse side effects very often.

RFG3F2 The side effects are terrible.

RFG3F1 Years ago, I had patches of not sleeping very well, and used to take a half a phenobarbitone, half a one just gave me a good night's sleep. Then I went for a long time and didn't need anything. Then I think I came here and I had a patch of sleeplessness, went to the doctor and he prescribed some tablets, which – he said he couldn't give phenobarbitone, they weren't allowed to prescribe them so I don't know what it was he gave me – but it gave me such an awful … it used to knock me out for two hours. And then I'd wake up wide awake, so I don't take them anymore. (Residential focus group 3, 1 male and two female participants, aged 75–85+)

Many of our focus group participants had not sought medical advice despite describing severe sleep problems because they did not want to be prescribed sleeping tablets and thought that this is what their doctor would offer. For these participants sleep problems were caused by lifestyle factors and although sleeping pills might be a remedy they were thought of as treating the symptom rather than the root cause of the problem. There was a general dislike of sleep medication, linked to the possibility of becoming addicted, the idea of sleeping pills being ‘unnatural’ and concerns that medicated sleep ‘won't feel like real sleep’. Echoes of this narrative can be seen in the exchange below between two paramedics. Both had had sleep problems in the past.

Moderator What have you used to help to sleep or keep awake?

ASFG2F3 Nothing.

Moderator Why not?

ASFG2F3 Well, it's only because I'm quite a natural. I like the natural complementary side of … I don't particularly like the allopathic way of treating your body. So I like things more natural and so that's why I definitely would never go to the doctor and say ‘Can I have some Zopiclone please.’ and I would never think about taking anything orally to make me either stay awake or go to sleep.

ASFG2M1 Not even any of the herbal type products?

ASFG2F3 No. (Ambulance Staff Focus group 2, 1 female and 1 male participant, aged 25–54)

Instead of taking hypnotics those drawing on this repertoire described their efforts to manage their sleep through non‐pharmacological means, including varying use of over‐the‐counter remedies, exercise, mediation, prayer, alcohol and other ‘personalised strategies’, similar to female respondents in a study by Hislop and Arber (2003). They criticised doctors for handing out sleeping pills too freely and, through their talk, depicted a moral image of themselves as exhibiting strength of character and ‘doing the right thing’ by resisting the medication. Although not seeing themselves as deserving patients who were in need of sleeping pills, they did see a role for pills in special circumstances. However, they expressed concerns about negative side effects of taking this type of medication and saw medicated sleep as being different from and inferior to natural sleep.

## Discussion

Through their talk, participants in our study depicted themselves and their relationships with hypnotics in a range of ways, drawing on different moral repertoires. Six repertoires as we have seen are evident – the ‘deserving’ patient, the ‘responsible’ user, the ‘compliant’ patient, the ‘addict’, the ‘sinful’ user and the ‘noble’ or virtuous non‐user. These repertoires clearly have much in common with the moral repertoires identified in other studies of medications use. Thus Lumme‐Sandt *et al*. (2000), in their study of the oldest old (aged 90 or over) in Finland, found that their respondents presented themselves as moral and responsible drug users by explaining the ‘objective reasons’ for their use, playing down the extent of such use and comparing it favourably with the assumed level of use by others. Similarly Dew *et al*. (2015), in their study of households in New Zealand, reported that respondents developed and articulated four distinct moral repertoires: a disordering society repertoire; a disordering self repertoire; a disordering substances repertoire and finally a re‐ordering substances repertoire. Our study has identified a broader variety of repertoires which have drawn on those described above but have honed them to legitimise the use or non‐use of hypnotic medications. In our study addiction, sin and virtue were more to the fore than in Lumme‐Sandt *et al*.'s (2000) study, while in comparison with Dew *et al*. (2015) we found a greater variety of repertoires relating to the ‘restoration of function’ (deserving user, compliant user). However, like Dew *et al*., we too found evidence of resistance to a disordered society (the virtuous non user), the disordering self (the addict) and the disordering substance (the responsible user). These differences may in part relate to the fact that we have focused on repertoires relating to a specific drug type – hypnotics.

While we have identified a range of distinctive moral repertoires in the data, in practice we found that participants orientated towards multiple repertoires which were often layered one on top of another. Use of these repertoires shifted over time and in response to what others revealed about their own pharmaceutical regimens and the moral discourse that was articulated around hypnotic use in the focus group. For example, the same person who initially presented themselves as a deserving patient claimed to be a responsible user and also a sinful user during subsequent discussion.

Each of the repertoires we have identified, we suggest, draws on the following crosscutting themes: addiction and control, ambivalence and reflexivity. Taking these in turn, it was common for our participants to attempt to distance themselves from sleeping pills by drawing on moralising talk, in recognition of the stigma around addiction and the associated imagery of illicit drug use. Concerns about addiction have long been reported in studies of benzodiazepine use (Gabe and Lipshitz‐Phillips 1984, North *et al*. 1995). Typically, participants were concerned about being (mis)represented as being an ‘addict’ and attempted to distance themselves from this image, describing strategies of self‐surveillance and self‐governance they had adopted in order to protect themselves from becoming addicted to sleeping pills. Addiction was in turn related to the issue of control; claiming to be in control challenged the idea that they were addicted. Hence participants emphasised how they were in control over whether they chose to take their medication or not, how many pills they took, how often they took them and their ability to stop taking such medications.

A second key theme was that of ambivalence, a response to medicine and medications which is said to be ever more common in late modern society where traditional authority and expertise are increasingly questioned (Nettleton 2006). Ambivalence towards sleeping pills was a prevailing theme across all the focus groups. For instance, participants articulating a compliant patient repertoire also acknowledged side effects and expressed doubts about the efficacy of their medication, regarding hypnotics as a ‘necessary evil’ (Gabe and Lipshitz‐Phillips 1982). At the same time those expressing strong anti‐medication sentiments could also see a role for sleeping pills in some circumstances. Some of this ambivalence may stem from the UK medical community's own ambivalent moral and political stance towards the pharmaceuticalisation of sleep problems. Although hypnotic medications are licensed for insomnia in the UK and doctors may see a role for them as a short term solution, they are viewed as far from perfect, being associated with various negative side effects. Furthermore, efforts have been made for some time to reduce hypnotic prescribing in UK primary care, measures which some of our participants at least, had experienced. These participants said their GP had advised about the risks of dependence and warned them that the drug should only be taken for a limited period. And yet it is clear from our respondents that it is still possible to obtain a prescription from some GPs and in some circumstances. Our data thus shed some further light on the ways in which processes of pharmaceuticalisation/ depharmaceuticalisation (Abraham 2011, Williams *et al*. 2011) work to shape the cognitive, cultural and affective framing of sleep problems. While long term hypnotic use continues to be commonplace in the UK it is now rarely embraced unquestioningly. Instead, it is reviewed in a critical and reflexive way, even by those who have been using this medication for a considerable period of time.

The final cross‐cutting theme was that of reflexivity. This was clearly demonstrated in the way that many of our focus group participants rejected the image of the passive consumer of medications. They preferred to engage reflexively with the different normative frameworks and discourses around medicine use to justify their own medication taking practices. They often seemed to act as ‘lay pharmacologists’ assessing the safety, efficacy and side effects of the drug’ (Webster *et al*. 2009). Like other studies (Dew *et al*. 2014), the participants in our study also drew on advice from various quarters, including medical advice, as and when they deemed it necessary, in developing personal medication practices. This reflects the social lives and meanings of medications, as medication taking practices become entangled in domestic routines and meanings within the therapeutic environment of the home and the competing forms of knowledge that co‐exist there. In domestic spaces these competing forms of knowledge can get mixed up or ‘hybridised’ (Dew *et al*. 2014). Consequently, forms of medical knowledge and clinical advice are reworked and reformed through the relationships people have with medications in daily life. As our analysis shows, responses to illness and wellbeing involve pragmatic decision‐making based not only on what works for people with sleep problems, but also on what they deem to be appropriate and acceptable in the context of their daily lives, rather than simply adhering to the rules set by medical experts. Thus some of our focus group members reported taking ‘drug holidays’ while others reduced the dose to see what the effect would be. In both cases this was done without consultation with a doctor. Amongst those invoking the repertoire of the virtuous non‐user, using alternative therapies to deal with sleep problems was often mentioned. All of these practices illustrate the desire of people to be reflexive about their medicines and reflect the nature of the late modern age where medical authority is no longer unquestionably accepted and lay experience if not expertise carries increasing weight (Williams and Calnan 1996).

At a broader level, our data therefore support conceptualisations of pharmaceuticalisation as being a dynamic bidirectional process, including various forms of expert patient/consumer resistance to pharmaceuticalisation. On the one hand, people may reject pharmaceuticals in the management of sleep problems and selectively alter therapeutic regimens in the home where pharmaceuticals may be used alongside or replaced by non‐pharmacological means of therapy. On the other hand, they may also present various challenges to GPs’ attempts (in line with current mandates) to reduce or restrict resorting to prescription hypnotics in primary care, through continuing to present themselves as deserving and in need of pharmaceuticals, questioning medical authority and knowledge and, on occasion, seeking prescription drugs outside the medical encounter, through practices such as sharing with friends, buying sleeping pills on the Internet and stockpiling medications for use at a later date.

Although documented cases of depharmaceuticalisation are rare, in the case of hypnotics to treat sleep problems at least, the process is best seen as being in a state of flux, particularly in the context of developments for more cognitive behavioural based forms of intervention, although doctors do not necessarily see Cognitive Behaviour Therapy for Insomnia (CBTi) as an alternative to medication. The degree to which sleep problems are subject to (de)pharmaceuticalisation in the future therefore remains uncertain and open to challenge.

## Notes

1 It was common for those who had been using hypnotics for several years to have used many different types and brands of medication. Of those who were taking prescription hypnotics at the time of the study (18), the majority (13) were taking Zopiclone or Zolpidem.

2 Xyrem has been included as it is prescribed to narcolepsy patients for the purpose of sedation and to help these patients consolidate their sleep at night. It also helps to reduce cataplexy during daytime.
